# Gesundheitliche Chancengleichheit in Krisenzeiten

**DOI:** 10.1007/s00103-023-03664-w

**Published:** 2023-02-21

**Authors:** Anke-Christine Saß

**Affiliations:** Abteilung für Epidemiologie und Gesundheitsmonitoring, Robert Koch-Institut, General-Pape-Str. 62–66, 12101 Berlin, Deutschland

„Verleih uns Frieden“, übertitelt der mitteldeutsche Komponist Heinrich Schütz das wohl bekannteste Stück seiner im Jahr 1648 gedruckten Sammlung „Geistliche Chormusik“. Der kreative Künstler, weitaus weniger bekannt als der 100 Jahre später geborene Johann Sebastian Bach, formuliert hier einen Wunsch, der aktueller nicht sein könnte. In Zeiten des Dreißigjährigen Krieges mit großem Leid, Zerstörungen und vielfältigen gesundheitlichen Folgen für die Bevölkerung schrieb er tröstliche und anrührende Musik. Auch heute, 350 Jahre nach seinem Tod, berührt sie viele Menschen. Im Jahr 2022, als die Beiträge dieses Heftes konzipiert und geschrieben wurden, gab es eine große Zahl von Konzerten, Festivals, Uraufführungen, Chorprojekten und Radiobeiträgen zum Gedenken an dieses Jubiläum. „Verleih uns Frieden zu unsern Zeiten“, so steht es im Text und passt so gut nach drei Jahren Pandemie und nachdem der russische Angriffskrieg gegen die Ukraine die Sicherheit in der Mitte Europas ein weiteres Mal infrage stellt.

Schon vor mehr als 350 Jahren, als Heinrich Schütz am Dresdner Hof tätig war, waren die Lasten der Verwerfungen der Zeit nicht gleich verteilt. Zur Biografie dieses Musikers gehört, dass er sich – heute würden wir sagen als Bessergestellter – als Musikdirektor mit auskömmlichem Einkommen für die ihm unterstellten Musiker der Hofkapelle und für die ihm anvertrauten Chorknaben einsetzte. Deren schwierige Lebensbedingungen in widrigen Zeiten beschrieb er mit klaren Worten und forderte seinen Dienstherrn, den sächsischen Kurfürsten Johann Georg I., zur Unterstützung auf.

In den letzten drei Jahren hat die COVID-19-Pandemie das Leben der Menschen in Deutschland und der Welt in ungeahnter und umfassender Weise verändert. Kaum ein Lebensbereich blieb dabei ausgespart. Manche Veränderungen erweisen sich als langfristig und zum Teil unumkehrbar. Branchenbezogene Einbrüche, Veränderungen in der Verteilung von Erwerbs- und Familienarbeit etc. beeinflussen langfristig Erwerbsbiografien, Lebensentwürfe und möglicherweise die Gesundheit. Die gesundheitlichen Langzeitfolgen von SARS-CoV-2-Infektionen sind derzeit Gegenstand vieler Studien. Der tatsächliche Anteil Betroffener kann noch nicht verlässlich geschätzt werden. Das gilt auch für die Belastungen, die dadurch für das Gesundheitssystem und die Gesellschaft entstehen werden. Auf der anderen Seite gab es auch positive Veränderungen in der Pandemie, wie zum Beispiel das breite Ausrollen neuer technischer Möglichkeiten für Kommunikation und Zusammenarbeit in der Arbeitswelt und die Etablierung von Homeoffice-Angeboten für viele Arbeitnehmerinnen und Arbeitnehmer. Die Bilanz nach drei Jahren „Ausnahmezustand“ fällt sicher für jede und jeden anders aus.

Und doch sind auch die Lasten der Verwerfungen unserer Zeit nicht zufällig oder gar gleichmäßig verteilt. In Politik und Gesellschaft ist inzwischen längst angekommen, dass es die vulnerablen, die besonders verletzlichen Bevölkerungsgruppen sind, die in der Pandemie besonders schwere Lasten tragen mussten – gesundheitlich, sozial, finanziell. Dieses Heft ist das erste von zwei Schwerpunktheften des *Bundesgesundheitsblattes*, die sich den Auswirkungen der COVID-19-Pandemie in den besonders vulnerablen Bevölkerungsgruppen widmen. Es reiht sich in die Serie „COVID-19 und Public Health“ mit bislang sechs Heften ein. Die Reihe startete im März 2021 mit einem Heft zu „Wissen, Einstellungen, Belastungen und Kommunikation in der Krise“. Danach folgten die Themen „Reaktionen des öffentlichen Gesundheitsdienstes“ (April 2021), „Epidemiologie“ (September 2021), „Gesundheit von Kindern und Jugendlichen“ (Dezember 2021) und „Impfungen“ (Dezember 2022).

In den beiden im Jahr 2023 erscheinenden Schwerpunktheften ist der Fokus auf diejenigen gerichtet, die in besonderer Weise von den negativen Folgen der Pandemie betroffen sind. Sie verzeichneten überdurchschnittlich hohe Infektionsraten, waren vermehrt von schweren Krankheitsverläufen betroffen oder leiden unter den Langzeitfolgen einer COVID-19-Erkrankung. Für einige Personengruppen war die Nicht-Inanspruchnahme von Versorgungsangeboten in der Pandemie besonders problematisch. Teile der Bevölkerung litten besonders unter den Eindämmungsmaßnahmen oder wurden durch Präventionsbotschaften und Impfangebote schlechter erreicht. Sehr unterschiedliche Personen/Personengruppen haben sich in der Pandemie als vulnerabel erwiesen. Im ersten Heft wird der Fokus auf Vulnerabilität aufgrund physischer Risiken durch Alter, chronische Krankheit und Behinderung gelegt. Im letzten Themenblock adressieren wir Schwangerschaft und Geburt als vulnerable Lebensphasen. Das zweite Heft, das im August 2023 erscheinen wird, enthält Beiträge zu Vulnerabilität aufgrund der sozialen Lage, u. a. mit Blick auf unterschiedliche Wohn- und Arbeitsbedingungen und ihren Einfluss auf die Gesundheit in der Pandemie.

Forschungsergebnisse zur Gesundheit älterer und alter Menschen in der Pandemie liegen inzwischen in großer Zahl vor. Diese Bevölkerungsgruppen standen in den letzten drei Jahren oft im Fokus der gesellschaftlichen Aufmerksamkeit. Das vielschichtige Thema nimmt im ersten Schwerpunktheft einen großen Raum ein. *Wünsche et al.* präsentieren Befunde aus dem bundesweiten Deutschen Alterssurvey (DEAS) und finden – zumindest für das erste Jahr der Corona-Pandemie – bei älteren Menschen in Privathaushalten im Vergleich zu jüngeren keine besonders ungünstige gesundheitliche Lage. Zur Gesundheit Hochaltriger in der Pandemie haben *Gerhards et al.* geforscht und festgestellt, dass in dieser Gruppe im Verlauf der COVID-19-Pandemie eine Zunahme psychischer Belastung zu beobachten ist. Die Situation in Alten- und Pflegeheimen beleuchten *Said et al.* aus dem Robert Koch-Institut. Sie berichten über Erfolge und Herausforderungen bei der Umsetzung infektionshygienischer Maßnahmen und beschreiben den Einfluss auf die Gesundheit der Bewohnenden. Im Beitrag von *Preuß et al.* wird die Lage in der ambulanten Pflege betrachtet. Die Analysen zeigen ein erhöhtes Erkrankungs- und Sterberisiko der Pflegebedürftigen im Vergleich zur Allgemeinbevölkerung. Zudem hat der pandemiebedingte Wegfall von Versorgungs- und Unterstützungsangeboten negative Auswirkungen auf die psychosoziale Verfassung der Gepflegten. *Wiegelmann et al.* berichten über „Deutschlands größten Pflegedienst“ – die pflegenden Angehörigen – in der Pandemie. Die Ergebnisse einer empirischen Erhebung weisen darauf hin, dass die Belastungen der Pflegenden in der Pandemie gestiegen sind und sich ihre Lebensqualität verschlechtert hat.

Neben dem Alter gelten verschiedene chronische Krankheiten als Risikofaktoren für eine SARS-CoV-2-Infektion bzw. für einen schweren Verlauf der Erkrankung. Aus Angst vor Ansteckung haben Menschen mit chronischen Krankheiten – darunter sind viele Ältere – Arzttermine in der Pandemie nicht wahrgenommen, auch von den Praxen wurden Termine abgesagt. Laut Hamburg City Health Study (HCHS) war jede/r 5. Patient/in mit einer chronischen Erkrankung vom Aussetzen medizinischer Leistungen betroffen. So zeigen es *Schäfer et al.* in ihrem Beitrag. Der Situation von Menschen mit Behinderungen in der COVID-19-Pandemie widmet sich *Habermann-Horstmeier*. Sie analysiert, welchen besonderen Risiken Menschen mit geistiger Behinderung ausgesetzt waren, etwa aufgrund ihrer Wohn- und Betreuungsform sowie von behinderungsassoziierten Gesundheitsfaktoren.

Der letzte Themenblock dieses Schwerpunktheftes springt thematisch noch einmal nach vorn, an den Beginn eines Lebens. Schwangerschaft, Geburt und ungewollte Schwangerschaft, wichtige Lebensereignisse für Frauen und ihre Partner/Angehörigen liefen in der Pandemie selten „ganz normal“ ab. Zum Beispiel gab es erstmals digitale Betreuungsangebote von Hebammen. Aus Sicht der Mütter, so beschreiben es *Bauer et al.* in ihrem Beitrag, sind diese passend und können die Betreuung in Präsenz sinnvoll ergänzen. Auch die Versorgung unter der Geburt war wegen des Infektionsrisikos zahlreichen Veränderungen unterworfen. *Batram-Zantvoort et al.* berichten von den Erfahrungen der Frauen im Kreißsaal in Zeiten pandemiebedingter Einschränkungen. Abschließend beschreiben *Maeffert und Tennhardt* wie es ungewollt Schwangeren in der COVID-19-Pandemie ging. Insbesondere für vulnerable Gruppen, z. B. Alleinerziehende, die ungewollt schwanger werden oder einkommensarme Frauen, ergaben sich zusätzliche Hürden für die Beratung und Versorgung.

Sehr viele Herausforderungen und viele Chancen für den Public-Health-Bereich, so kann man die letzten drei Jahre vielleicht zusammenfassen. Das spiegelt sich auch in den unterschiedlichen Beiträgen dieses Heftes wider, die alle um das Thema Public Health für vulnerable Gruppen kreisen. Die meisten Beiträge greifen dabei auf Studiendaten und wissenschaftliche Ergebnisse aus den Jahren 2020/2021 zurück. Wenige Daten wurden später erhoben. Dieses Heft ist also nur ein Zwischenstand zum Thema „COVID-19 und Public Health: Menschen mit besonderen Risiken und Versorgungsbedarfen“. Über die mittel- und langfristigen Folgen der Pandemie – Art, Ausmaß, besonders betroffene Gruppen – gibt es derzeit nur Vermutungen.

Als Lessons Learned aus drei Jahren Pandemie möchten viele nun die Chancen der pandemiebedingten Veränderungen nutzen (z. B. mit Blick auf digitale Versorgungsangebote), deutlich hervorgetretene Probleme im Public-Health-Bereich angehen und Neuanfänge wagen. Dazu gehört auch, die besondere Betroffenheit verletzlicher Bevölkerungsgruppen in Krisen anzuerkennen, ihre Situation in der Pandemie zu analysieren und Wege für Verbesserungen zu suchen. Das führt zurück zu Heinrich Schütz und seiner Musik. Unabhängig davon, ob man religiös ist oder nicht (Heinrich Schütz war es als Kind seiner Zeit und Lebensorte natürlich), eint der Wunsch nach einem guten Leben, in Frieden und Sicherheit. Wenn es uns gelingt, Lehren aus der Pandemie zu ziehen und gesundheitliche Chancengleichheit für alle Bevölkerungsgruppen zu fördern, tragen wir dazu bei. Ein gleichwertiger Zugang zu gesunden Lebensweisen und medizinischer Versorgung für alle Gruppen der Bevölkerung bleibt ein vorrangiges Public-Health-Ziel.

Ein herzlicher Dank gebührt Frau Dr. Anke Spura (BZgA) für ihre Impulse bei der Konzeption dieses Heftes.

